# Neurodevelopmental Outcome in Very Preterm Infants Randomised to Receive Two Different Standardised, Concentrated Parenteral Nutrition Regimens

**DOI:** 10.3390/nu15224741

**Published:** 2023-11-10

**Authors:** Colin Morgan, Samantha Parry, Julie Park, Maw Tan

**Affiliations:** 1 Liverpool Women’s Hospital, Liverpool L8 7SS, UK; 2 Alder Hey Children’s Hospital, Liverpool L14 5AB, UK

**Keywords:** parenteral nutrition, neurodevelopment, preterm, protein, energy, hyperglycaemia, insulin, hypophosphataemia, hypokalaemia

## Abstract

We have previously shown that increasing parenteral protein (target: 3.8 versus 2.8 g/kg/d) and energy (12% versus 10% glucose; 3.8 versus 2.8 g/kg/d) intake using a Standardised, Concentrated with Added Macronutrients Parenteral (SCAMP) nutrition regimen ameliorates early head growth failure in very-preterm infants (VPIs). We hypothesised that the SCAMP nutrition regimen would also improve neurodevelopmental outcome. The original double-blind randomised, controlled study (ISRCTN: 76597892) received ethical approval. VPIs were randomised to either start SCAMP or remain on the control regimen. The consent process included neurodevelopmental assessments (Bayley III), all of which were performed (blinded) at 2–3.5 years of corrected gestational age. Bayley III assessments were performed for 38/60 SCAMP survivors and 41/63 control survivors at means of (sd) 29.2 (3.7) and 20.0 (3.9) months, respectively. Motor, cognitive, language, and combined scores were all higher in the SCAMP intervention group, but none of the differences were statistically significant. Nutrient intake and biochemical monitoring data confirmed that protein/energy ratios were maintained in the SCAMP intervention group without increasing the incidence of hyperglycaemia, insulin treatment, or the derangement of plasma mineral/electrolyte levels. This study did not show a statistically significant improvement in neurodevelopmental outcome when administering higher parenteral protein/energy intakes despite optimal energy and mineral intakes.

## 1. Introduction

The risk of an adverse neurodevelopmental outcome among very-preterm infants (VPIs) remains high. This group of infants is also vulnerable to postnatal growth failure in the first few weeks of life, and there is increasing evidence that this is amenable to improved nutrition [1,2]. The pattern of growth failure in infants born <30 weeks was described in detail by Ehrenkranz [3], who demonstrated that growth in head circumference (HC) was also insufficient to match the corresponding fetal reference curves [3]. This results in a growth curve shifting away from the original centile and a falling standard deviation score (SDS) in the early postnatal period. The lowest SDS occurs at about 4 weeks postnatal age [4,5] for VPIs. Typically, there is a period of later catch-up head growth, but the deficit persists after 36 weeks corrected gestational age (36wCGA) [6]. This effect is more marked among infants born <26 weeks gestation [7]. Head growth is an important measure of growth failure because it correlates with brain weight and volume, as shown in post-mortem studies [8] and through neuroimaging at term [9]. Brain growth between birth and the expected date of delivery is a key predictor of long-term brain growth [10,11]. In VPIs, lower rates of postnatal head growth are associated with poorer developmental scores and higher rates of cerebral palsy at 18 months [12]. Hack showed that a subnormal head size at 8 months was predictive of poorer verbal and performance IQ scores at 3 [13] and 8 [14] years of age.

Improving early protein and energy intake in VPIs in the first few weeks of life has been associated with improved neurodevelopmental outcomes at 18 months. Stephens et al. [15] found that nutritional intake in the first week of life was particularly important. The impact of heterogeneity (both nutritional interventions and neurodevelopmental assessments) has been recognised as a major limiting factor in interpreting the evidence base [16]. VPIs are dependent on neonatal parenteral nutrition (NPN), especially in the first 14 days of life, to sustain sufficient nutrient intake while milk feeds are being established. There have not been any RCTs comparing a combined parenteral protein (in the form of intravenous amino acids (AA)) and energy intervention with neurodevelopmental assessment as a primary outcome measure. In addition, increasing parenteral protein and energy has potential metabolic consequences such as an increased risk of hyperglycaemia [17] and so-called “refeeding syndrome” [18]. Both are associated with adverse preterm outcomes. Any standardized nutritional strategy that increases protein and energy intake will therefore need to include guidance on addressing the increased risk of metabolic derangement.

We have previously reported that increasing parenteral protein and energy intake using a Standardised, Concentrated with Added Macronutrients Parenteral (SCAMP) nutrition regimen ameliorates early head growth failure at 28 days and 36 weeks corrected gestational age (CGA) in VPIs [19]. The effect was greatest in infants at less than 27 weeks of gestation. We hypothesised that the SCAMP nutrition regimen would improve neurodevelopmental outcomes. **Aim**: We sought to compare neurodevelopmental outcomes, measured using Bayley III assessments at 2–3.5 years of age, in VPIs randomised to receive SCAMP nutrition (12% glucose, maximum 3.8 g/kg/day protein/lipid) or a control standardised, concentrated PN regimen (10% glucose, maximum 2.8 g/kg/day protein/lipid).

## 2. Methods

This study (ISRCTN: 76597892) received ethical and regulatory approval and is described in detail herein, including the published primary outcome [19]. Neurodevelopmental assessment was a planned secondary outcome. The original study recruited infants born at <29 weeks gestation, weighing <1200 g, and admitted to the Neonatal Intensive Care Unit (NICU) at Liverpool Women’s Hospital (LWH) within 48 h of birth between October 2009 and July 2012. This was a single-centre RCT (Randomised Controlled Trial) with blinding applied to all those involved in care and assessment except for the dispensing pharmacist. Permission to be approached for later neurodevelopmental follow-up was part of the original consent process. Randomisation occurred within 120 h, and infants were stratified into two groups: <27 weeks gestation and between 27 + 0 and 28 + 6 weeks gestation [20]. Infants unlikely to survive the first week of life or suffering from major congenital gastrointestinal or neurological anomalies were excluded [20]

The original study intervention compared the standardised Concentrated Additional Macronutrients Parenteral nutrition regimen with the contemporary standardised, concentrated neonatal PN (control) regimen [20]. The control regimen started as soon after birth as possible and was incrementally increased to provide a maximum of 2.8 g/kg/protein (3.3 g/kg/d AA) and 85 kcal/kg/d energy after day 4. All study infants received the control PN regimen initially and, following consent, were randomised to either start SCAMP regimen (designed to provide 3.8 g/kg/d of protein or 4.3 g/kg/d of AA and 105 kcal/kg/d after day 4) or continue receiving the control regimen. The regimens have been described in detail previously [19,20], and both used Vaminolact (Fresenius-Kabi, Runcorn, UK) as the parenteral AA source and Intralipid 20% (Fresenius-Kabi) as the parenteral lipid source. Importantly, the regimen includes a protocol of standardised electrolyte/mineral supplementary infusions to correct one or more deficiencies that may arise despite administering daily maintenance. The treatment thresholds for hypokalaemia and hypophosphatemia were 3.0 mmol/L and 1.4 mmol/L, respectively. Infants with hyperglycaemia were also subject to an insulin treatment protocol, with treatment being initiated when two consecutive blood glucose measurements >12 mmol/L were obtained.

All infants received clinical care in accordance with LWH NPN protocols, including fluid management; introducing, increasing, and stopping enteral feeds; and biochemical monitoring. The study intervention continued until 28 completed days of life. PN was discontinued once enteral feeds exceeded 75% total. The transition from PN to enteral feeds has been described previously [20] and involved the preferential use of expressed or donor breast milk, which remained unfortified until 150 mL/kg/day enteral feeds were administered. Patient data were collected from electronic patient records. Daily enteral and parenteral protein and energy intake data were calculated as described previously. Biochemical (including blood gas) monitoring, electrolyte/mineral supplementation, and insulin treatment data were also collected from an electronic patient data system. Mean daily blood glucose was estimated from the intermittent blood-glucose-monitoring regime that was part of routine clinical care.

Parents were contacted after their children had reached 2 years of corrected gestational age to make arrangements for a Bayley III neurodevelopmental assessment to be performed in their own homes whenever possible. The aim was to perform assessments at 30 months CGA using one of two assessors (MT or SP). When home assessment was not possible, the assessors used local outpatient clinics. The raw scores of each subtest were converted to scaled and composite scores. A composite score of 100 is equivalent to normative mean. The combined score is a cognitive/language average validated to offer comparison with the Mental Developmental Index. Bayley III cognitive, language, and motor composite scores were calculated along with the combined composite score as described by Johnson et al. [21].

### Analytical Methods

Data were analysed using the same general approach as described for the primary outcome [19]. Thus, following primary analysis, developmental outcomes were analysed via a general linear model controlling for stratum, using sensitivity analyses that included covariates to adjust for potentially important group imbalances (birthweight, sex, and nutrient intake) and taking multiple births into account. T-tests were used for continuous variables, and Fisher’s exact test analyses were used for categorical variables.

Mean daily blood glucose data were collected from intermittent blood gas measurements adjusted for the variable frequency and timing, as previously described [22].

Plasma mineral and electrolyte data comprised the morning sample taken as part of a daily biochemical monitoring protocol for infants receiving PN. Missing daily data were replaced with either an average value (between the preceding and subsequent measurements) or a repeat sample (if the original was inadequate for analysis). Outlier measurements were excluded only if there were blood gas trend data (potassium and calcium only) to confirm a measurement was unlikely to be valid. The resulting missing value was then replaced as described.

## 3. Results

The original study randomised 150 infants to SCAMP (n = 74) and control (n = 76) groups. Bayley III assessments were performed for 38/60 SCAMP survivors (3 refused consent, 15 were uncontactable, and 4 were unable to proceed to the follow-up process) and 41/63 control survivors (2 refused consent, 13 were uncontactable, and 7 were unable to proceed to follow-up process). Table 1 summarises the demographic, nutritional intake, and primary outcome data from the original study and the same data for those infants that had been undergoing neurodevelopmental assessment.

This shows that this subgroup of SCAMP and control infants closely matches the demographic, protein and energy intake, and head growth data of the original study survivors. The nutrition data also confirm that although the study intervention lasted 28 days, most of the increases in protein and energy intake were achieved in the first 14 days of life.

**Figure 1:**
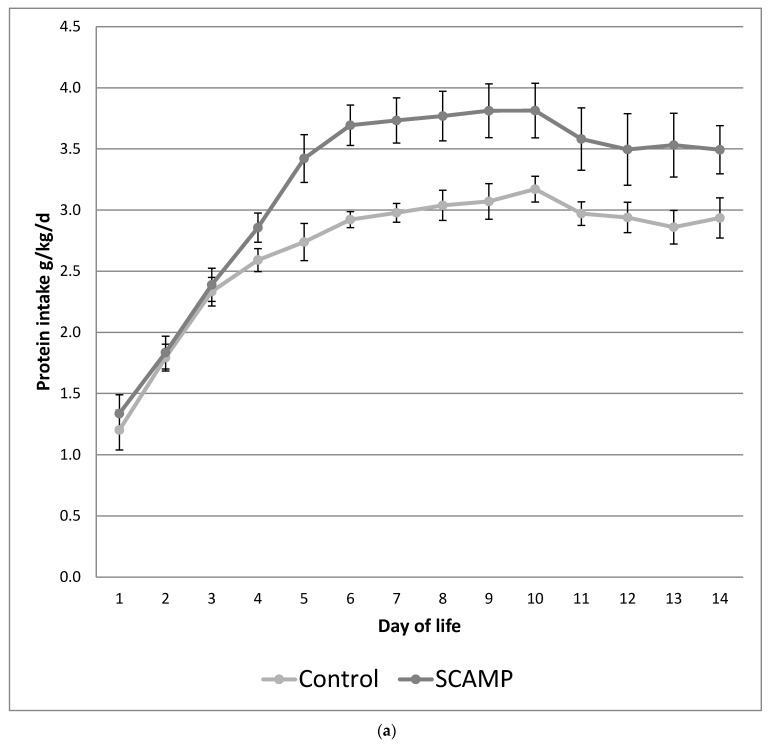

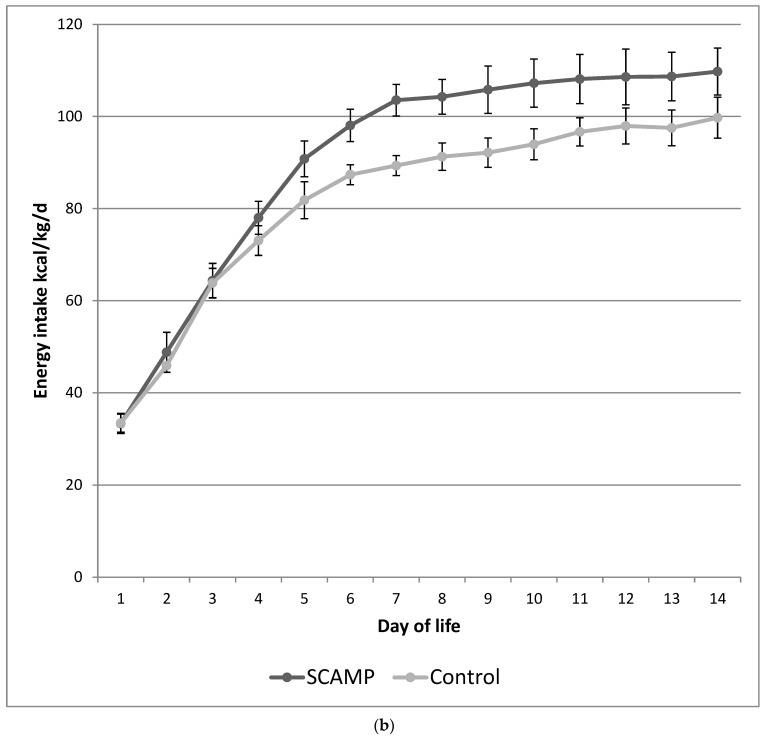
(**a**) Mean daily protein intake (g/kg/day) over the first 14 days of life in SCAMP versus control infants undergoing neurodevelopmental follow up. Error bars represent 95% confidence intervals for the mean. (**b**) Mean daily energy intake (kcal/kg/day) over the first 14 days of life in SCAMP versus control infants undergoing neurodevelopmental follow up. Error bars represent 95% confidence intervals for the mean.

Figure 1a,b illustrate the differences in the daily protein and energy intake in the SCAMP and control groups over the first 14 days of life. The protocol ensured an incremental introduction of PN so that maximum parenteral intakes were not achieved until after day 4. This design resulted in no difference in protein and energy intake in the first 3 days of life. The mean differences (95% confidence interval) were 0.53 g/kg/day of total protein and 9 kcal/kg/day of total energy over the first 14 days of life.

Bayley III cognitive, language, and motor composite scores together with the calculated combined composite score are shown in Table 2. There were no differences in the age of assessment. Motor, cognitive, language, and combined scores were all higher in the intervention population, but none of the differences were statistically significant. The differences were even greater in the lower-gestation-age stratum but, again, not statistically significant. Table 3 shows that the proportion of infants scoring < 85 is lower for all composite outcomes in the SCAMP group, and there were no statistically significant differences after correcting for multiple comparisons (Bonferroni method). A model controlling for birthweight, sex, protein and energy intake, and clustering (due to multiple births in the sample) yielded almost identical results.

**Figure 2:**
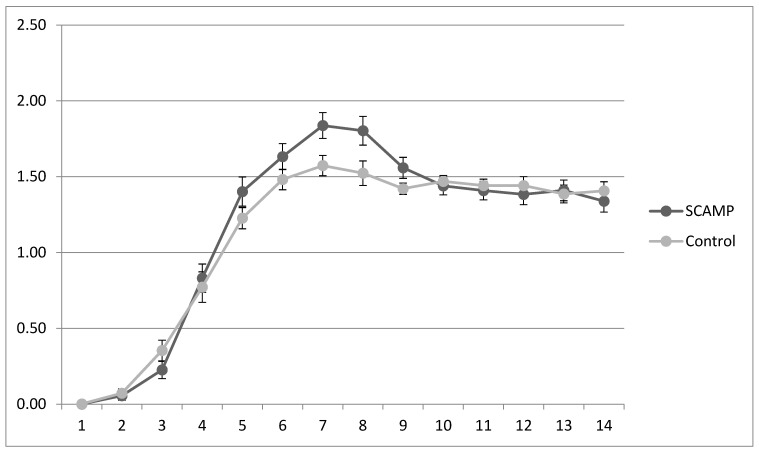
Mean (sd) daily plasma phosphate levels (mmol/L) in SCAMP and control groups over the first 14 days of life.

Table 4 shows the mean potassium, phosphate and calcium intake, and plasma data over the first 14 days of life. There are no differences in intakes or plasma levels. However, the infants receiving SCAMP required double the number of supplementary potassium and phosphate infusions compared to the controls. This supplementation occurred primarily during the period of maximum protein intake: 83% potassium supplementation occurred between days 3 and 8, and 82% phosphate supplementation occurred between days 4 and 9 (daily phosphate intake shown in Figure 2). This pattern was exhibited by the subgroup undergoing neurodevelopmental assessment and the 24–26-week stratum wherein the supplementation rates were higher.

**Figure 3:**
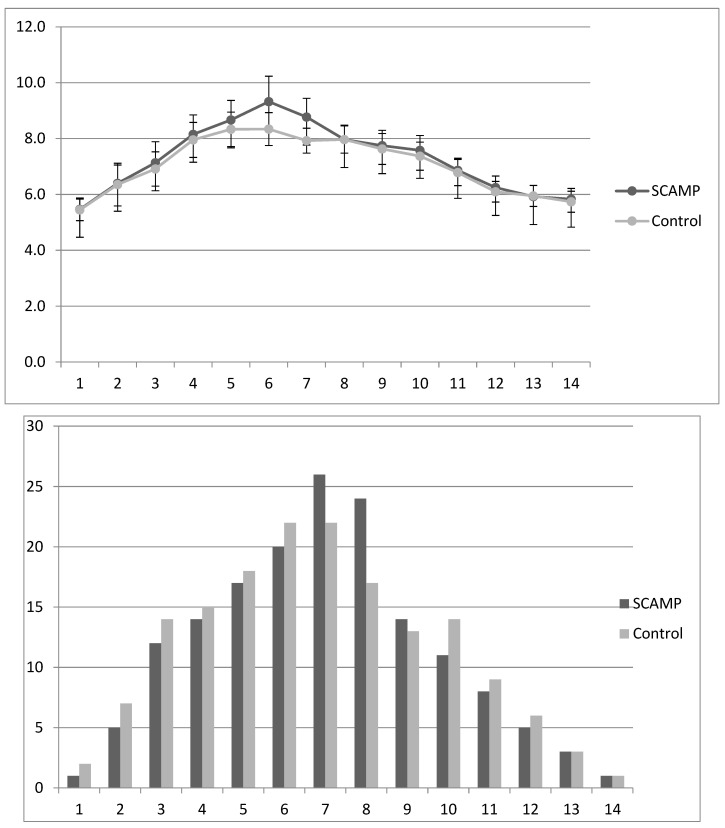
Mean (sd) daily blood glucose levels (mmol/L) in SCAMP and control infants over days 1–14, with corresponding bar chart describing percentage of infants receiving any amount of insulin during each of the first 14 days of life.

Table 5 shows that there was no difference in mean daily blood glucose (mmol/L) in the first 14 days of life, although there was a difference on day 6 (the peak mean daily blood glucose level) of borderline statistical significance. Insulin supplementation was only marginally higher in the SCAMP group (not statistically significant). The details of this finding are shown in Figure 3. In those that received insulin, the median (IQR) total dose received was 5.4 (2.7–11.7) versus 7.7 (2.2–13.9) IU/kg in the SCAMP and control groups, respectively. Again, the subgroup undergoing neurodevelopmental assessment reflected these findings, and the 24–26-week stratum showed higher insulin treatment rates without a difference between the groups.

## 4. Discussion

The study demonstrates that there were higher motor, cognitive, language, and combined Bayley III scores at 30 months when comparing SCAMP and control infants. However, none of these findings were statistically significant, and a larger study with power to assess neurodevelopmental outcomes is required. The importance of linking early anthropometric data, particularly head growth, and later neurocognitive outcomes has been previously highlighted [23,24] and is an important feature of this work. The differences in neurodevelopmental outcome were greater in the lower-gestation-age stratum (although still not statistically significant), matching the primary outcome results (i.e., change in head growth in the first 28 days of life) reported previously [19]. Early postnatal head growth is a predictor of later neurocognitive outcomes [23,24], but this relationship is not fully understood, and the precise period of sensitivity to head growth failure may vary in different preterm populations. This is important when developing nutritional interventions to improve early postnatal head growth. The results of this study may provide information about how to design future NPN studies with the power to investigate neurodevelopmental outcome as the primary outcome measure. The key findings and advantages of this study are as follows:

1. This study design ensured parenteral protein and energy intakes were incrementally introduced from birth so that the intervention did not achieve higher protein and energy intakes until day 4;
2. There are potentially greater effects of the intervention in the 24–26-week stratum;
3. Hypophosphatemia and hypokalaemia were avoided during hyperalimentation via higher supplementation rates in the SCAMP population;
4. The higher glucose intake in the SCAMP infants did not result in significantly higher rates of hyperglycaemia or insulin use when compared to the controls.

There are recent NPN RCTs that have compared standard and high parenteral protein (amino acid) intakes and reported neurodevelopment as a secondary outcome. Blanco [25] and Burratini [26] found no benefits using incremental early and high-dose parenteral amino acid regimens for 7 and 10 days, respectively. Balakrishnan [27] compared a control incremental AA regimen with a 4 g/kg/day target (similar to the SCAMP intervention regimen in this paper) via an intervention that started the administration of high doses from birth. There were no benefits for neurodevelopment nor negative effects on head growth. These findings are consistent with those from other studies examining immediate high-dose AA administration from birth [28]. None of these studies increased energy intake with increases in AA intake as was performed in the current study. In a secondary analysis of a large RCT, Poindexter [29] showed early AA administration resulted in better head growth at 36 weeks CGA and fewer infants with suboptimal head growth at 18 months, but no differences in neurodevelopmental outcome were observed. The group receiving early AA intake also had higher early energy intakes.

It is possible that high-dose AA administration without a sufficient energy intake at birth or during the first week of life may lead to high plasma AA levels, excessive AA oxidation, or other metabolic complications harmful to growth [30]. Suboptimal NPN AA formulations may aggravate this problem [31]. Incremental increases in both amino acid and energy levels simultaneously, as in this study (Figure 1a,b), may allow for more effective utilisation of AA for growth. Tan et al. [32] performed a study using incremental increases in both parenteral protein and energy with a standard (control) and a high target. Enteral intakes were also different. Neurodevelopmental outcome showed no differences at 9 months, but both the control and intervention groups failed to achieve their intended nutritional targets. Nevertheless, protein and energy deficits were correlated with early head growth [33]. In a non-randomised study, Cormack also demonstrated improved head growth with higher parenteral and enteral protein/energy intakes [2]. A systematic review of high versus low parenteral amino acid intakes highlighted the different impacts of isocaloric versus increased calorie intakes on growth outcomes [34]. This review suggested that failure to provide adequate energy when increasing parenteral protein intake may fail to benefit head growth and may even cause potential harm. This concern has received further validation following the publication of the ProVIDE study, where isocaloric increases in parenteral amino acid dose failed to benefit growth [35] or neurodevelopmental outcome [36].

Increasing parenteral energy intake has potential consequences. Increasing glucose intake as part of increasing overall energy intake has the potential to increase the risk of hyperglycaemia. Hyperglycaemia is associated with an increased risk of many preterm morbidities, including major cranial ultrasound abnormalities and neurodisability [37,38]. The original SCAMP nutrition study reported no difference in severe intraventricular haemorrhage, periventricular leukomalacia, or other major preterm morbidities when comparing the corresponding control and intervention groups [19]. Insulin, used as a treatment to prevent hyperglycaemia, has also been associated with adverse neurodevelopmental outcomes, but this association was made using a much lower treatment threshold and target mean blood glucose than described in this study [39,40]. Much-lower-quality studies using insulin to treat hyperglycaemia provide much-lower-quality evidence, not least because of large variations in treatment thresholds, protocols, and monitoring [17,41]. We have previously reported that parenteral amino acid intakes reduce the risk of both hyperglycaemia and insulin treatment, and this may in part account for the similar mean blood glucose profiles and frequency of insulin treatment seen for the SCAMP and control infants [23,42,43]. Our data suggest insulin supplementation can be safely used at a (high) treatment threshold of 12 mmol/L to facilitate higher energy intakes without increasing the risk of neurodevelopmental problems, but this needs further investigation.

Improving growth by increasing parenteral amino acid intake not only requires adequate energy intakes but also requires additional electrolytes (primarily potassium) and minerals (particularly phosphate). These increased requirements have been well described and are sometimes referred to as refeeding syndrome: a triad of hypokalaemia, hypophosphatemia, and hypercalcaemia [18,44,45]. Inadequate supplementation has resulted in hypophosphatemia in the intervention groups of trials investigating increased parenteral amino acid supplementation. This has been associated with an increased risk of sepsis [46], adverse neurodevelopmental outcomes, and mortality [45]. We have shown that additional potassium and phosphate supplementation is indeed required to support increased protein and energy intakes, and this approach removes the additional risk of hypophosphatemia and hypokalaemia. We suggest that parenteral amino acid supplementation should not be considered as an isolated intervention that risks refeeding syndrome but that normal physiology requires adequate energy, potassium, and phosphate as part of the nutritional package needed for healthy growth. Failure to ensure this in previous studies may explain why enhanced amino acid intake has not translated into benefits for growth or neurodevelopmental outcome.

Comparing nutritional interventions and outcomes is difficult when there is such variation in parenteral/enteral interventions, methodologies for growth/developmental assessment, and other laboratory measures [16]. There has been a call for more uniform reporting [47]. NPN standardisation, used in this study, may help reduce unintended variation in nutritional intakes and induce greater protocol compliance [19,28]. This is important when apparently small differences in study protocols (such as the starting dose or incremental rates of AA and/or energy) lead to different growth and/or neurodevelopmental outcomes. Reducing unintended inter-patient variation in Nutritional intakes may allow previously unrecognised differences in patient group responses, such as gestation or gender, to be identified. Males have higher calorie requirements in later life, and the association of early growth failure with poorer neurodevelopmental outcomes was greater in preterm males than females in a large population-based study [48]. There is evidence from cohort studies that increasing parenteral protein and energy intake improves neurodevelopmental outcome among boys but not girls [49]. This phenomenon is well described in RCTs regarding the protein and energy supplementation of preterm infants provided in enteral feeding studies [50,51], but we did not find any evidence that males were at higher risk of adverse neurodevelopmental outcomes in this study.

The main weaknesses of this study are that neurodevelopmental outcome was a planned secondary outcome and that we only had the power to assess early head growth. There are also missing data resulting from deaths and loss to follow-up. There is consistent evidence in the literature that the cognitive, language, and motor composites of the Bayley III scales underestimate the proportion of children with developmental delay when using the conventional cut off score of 70 [52,53]. We used the corrective approach described by Johnson et al. [21], although other, albeit similar, methods of correction have since been described [54]. It has been suggested that all these approaches have limitations [55], adding to the potential weaknesses of this study. The two assessors were both trained in Bayley III, with the aim of supporting neurodevelopmental follow-ups in preterm research, but individual variation between the assessors was not specifically evaluated in this study. Nevertheless, the population undergoing neurodevelopmental assessment was a close representation of the original study in terms of characteristics upon incorporation into the study, nutritional intake, and growth outcome measurements. The complexity of the intervention (involving increases in parenteral protein, lipid, and glucose intake) complicates the interpretation of the findings. However, the study is consistent with the growing body of evidence suggesting that increasing AA supplementation without additional energy is not beneficial for growth or neurodevelopmental outcomes.

## 5. Conclusions

This study showed higher Bayley III scores (none of which were statistically significant) after administering increased target parenteral protein and energy intake with an incremental increasing regimen in the first 4 days of life. These data are consistent with the previously published benefits for early head growth. Powering future nutritional studies designed to assess neurodevelopmental outcome requires consideration of the wider physiological implications of parenteral amino acid supplementation, particularly the need for adequate energy and potassium/phosphate supplementation.

## Figures and Tables

**Table 1:** Demographic, nutrient intake, and POM (primary outcome measure) data comparing original study population with follow-up population. Data are expressed as means (standard deviation). PN: parenteral nutrition; OFC: Occipitofrontal head circumference; SDS: standard deviation score.

	Original RCT			Neurodevelopmental FU (Follow up)		
Demographics	SCAMP (n = 74)	Control (n = 76)	*p*	SCAMP (n = 38)	Control (n = 41)	
Birthweight (g)	900 (158)	884 (183)		925 (152)	900 (185)	
Birthweight (SDS)	−0.47 (0.79)	−0.47 (0.73)		−0.41 (0.83)	−0.53 (0.81)	
Gestation (weeks)	26.8 (1.3)	26.6 (1.4)		27.0 (1.2)	26.8 (1.3)	
Sex (male)	44 (59.5%)	39 (51.3%)		22 (58%)	24 (59%)	
Nutrient intake	SCAMP (n = 66)	Control (n = 69)		SCAMP (n = 38)	Control (n = 41)	
Total protein d1–14 (g/kg/d)	3.20 (0.28)	2.68 (0.24)	**<0.001**	3.21 (0.32)	2.68 (0.18)	**<0.001**
PN protein d1–14 (g/kg/d)	2.82 (0.51)	2.26 (0.42)	**<0.001**	2.81 (0.55)	2.24 (0.40)	**<0.001**
Total energy d1–14 (kcal/kg/d)	91 (7)	81 (8)	**<0.001**	91 (7)	82 (9)	**<0.001**
PN energy d1–14 (kcal/kg/d)	76 (14)	64 (8)	**<0.001**	76 (14)	64 (12)	**<0.001**
Total protein d15–28 (g/kg/d)	3.18 (0.49)	3.08 (0.56)	**0.24**	3.19 (0.44)	3.20 (0.42)	
PN protein d15–28 (g/kg/d)	1.09 (1.20)	0.85 (1.03)	**0.20**	1.20 (1.17)	0.75 (0.85)	
Total energy d15–28 (kcal/kg/d)	112 (15)	109 (19)	**0.24**	111 (15)	113 (14)	
PN energy d15–28 (kcal/kg/d)	31 (33)	24 (29)	**0.16**	35 (33)	21 (24)	
Primary outcome measure	SCAMP (n = 66)	Control (n = 69)		SCAMP (n = 38)	Control (n = 41)	
OFC SDS (randomisation)	**See reference** [19]			−1.57 (0.82)	−1.50 (0.68)	
ΔOFC 28 days (POM)	**See reference** [19]			31 (9)	26 (8)	
ΔOFC SDS 28 days (POM)	**See reference** [19]			0.04 (0.61)	−0.27 (0.63)	
OFC SDS 36 weeks CGA	**See reference** [19]			−0.91 (1.12)	−1.31 (1.25)	

**Table 2:** Mean (sd) Bayley III composite scores in SCAMP versus control groups (24–26-week gestation stratum is shown separately).

Neurodevelopmental FU (All Infants)	SCAMP (n = 38)	Control (n = 41)	Mean Difference (95% CI)	*p*-Value
Corrected age (months)	29.2 (3.7)	30.0 (3.9)		**0.49**
Combined composite score	84 (15)	78 (14)	6 (−0.8 to 12)	**0.09**
Cognitive composite score	87 (15)	81 (14)	6 (−0.5 to 12)	**0.08**
Language composite score	81 (18)	76 (17)	6 (−2 to 13)	**0.11**
Motor composite score	79 (13)	76 (15)	3 (−4 to 9)	**0.38**
Neurodevelopmental FU (Gestation 24–26 weeks)	SCAMP (n = 15)	Control (n = 16)	Mean Difference (95% CI)	*p*-value
Corrected age (months)	28.9 (3.4)	30.3 (3.5)		**0.32**
Combined composite score	84 (15)	78 (14)	8 (−3 to 19)	**0.14**
Cognitive composite score	85 (17)	75 (16)	10 (−2 to 21)	**0.11**
Language composite score	75 (20)	67 (13)	7 (−5 to 19)	**0.25**
Motor composite score	74 (15)	69 (18)	5 (−7 to 17)	**0.44**

**Table 3:** Number of infants (%) with Bayley III composite scores < 85 in SCAMP versus control groups (24–26-week gestation stratum is shown separately).

Neurodevelopmental FU	All Infants			Gestation 24–26 Weeks		
	SCAMP (n = 38)	Control (n = 41)	*p*	SCAMP (n = 15)	Control (n = 16)	*p*
Corrected age (months)	29.2 (3.7)	30.0 (3.9)	**0.49**	28.9 (3.4)	30.3 (3.5)	**0.32**
Combined score < 80: n (%)	11 (29)	21 (51)	**0.07**	6 (40)	11 (69)	**0.15**
Cognitive score < 85: n (%)	11 (29)	18 (47)	**0.17**	4 (27)	8 (50)	**0.27**
Language score < 85: n (%)	16 (42)	29 (71)	**0.013**	9 (60)	16 (100)	**<0.01**
Motor score < 85: n (%)	21 (55)	27 (66)	**0.36**	11 (73)	10 (63)	**0.70**

**Table 4:** Original SCAMP and neurodevelopmental follow-up cohort: mean (sd) potassium, phosphate and calcium intake (mmol/kg/d), and plasma levels (mmol/L) over days 1–14 along with the number of infants (%) receiving at least 1 supplementary infusion over the same period.

Mineral/Electrolyte	Original RCT			Neurodevelopmental FU		
	SCAMP (n = 74)	Control (n = 76)	*p*	SCAMP (n = 38)	Control (n = 41)	*p*
Mean potassium intake	1.48 (0.30)	1.57 (0.29)		1.49 (0.26)	1.65 (0.19)	
Mean phosphate intake	1.21 (0.31)	1.15 (0.21)		1.20 (0.27)	1.15 (0.21)	
Mean calcium intake	0.89 (0.27)	0.93 (0.25)		0.89 (0.21)	0.98 (0.27)	
Plasma potassium level	4.79 (0.51)	4.87 (0.57)		4.84 (0.44)	4.99 (0.56)	
Plasma phosphate level	1.66 (0.19)	1.71 (0.24)		1.65 (0.16)	1.74 (0.26)	
Plasma calcium level	2.16 (0.15)	2.20 (0.15)		2.17 (0.13)	2.21 (0.13)	
Potassium supplement: n (%)	38 (51)	18 (24)	**0.0007**	15 (39)	8 (20)	**0.08**
Phosphate supplement: n (%)	54 (73)	36 (47)	**0.0016**	27 (71)	18 (44)	**0.023**
Calcium supplement: n (%)	8 (11)	6 (8)	**0.59**	4 (11)	2 (5)	**0.42**
Infants at 24–26 wks gestation	SCAMP (n = 35)	Control (n = 36)	*p*	SCAMP (n = 15)	Control (n = 16)	*p*
Potassium supplement: n (%)	21 (60)	11 (31)	**0.017**	7 (47)	2 (13)	**0.053**
Phosphate supplement: n (%)	32 (91)	18 (50)	**0.0002**	13 (87)	8 (50)	**0.053**
Calcium supplement: n (%)	3 (9)	3 (9)	**1.0**	4 (27)	2 (13)	**0.39**

**Table 5:** Original SCAMP and neurodevelopmental follow-up cohort: mean (sd) daily blood glucose (mmol/L) over days 1–14 and day 6 along with the number of infants (%) receiving at least 1 insulin infusion over the same period.

Insulin/Glucose	Original RCT			Neurodevelopmental FU		
	SCAMP (n = 74)	Control (n = 76)	*p*	SCAMP (n = 38)	Control (n = 41)	*p*
Mean blood glucose (d1–14)	7.50 (1.80)	**7.16 (1.71)**	0.24	7.32 (1.66)	7.02 (1.72)	**0.43**
Mean blood glucose (d6)	9.49 (3.76)	**8.37 (2.46)**	0.037	9.66 (4.47)	8.24 (2.84)	**0.093**
**Insulin use**	**39 (53)**	**33 (44)**	**0.33**	**16 (42)**	**18 (44)**	**1.0**
**Infants at 24–26 wks gestation**	**SCAMP (n = 35)**	**Control (n = 36)**	***p***	**SCAMP (n = 15)**	**Control (n = 16)**	***p***
Mean blood glucose (d1–14)	8.27 (1.67)	**7.86 (1.67)**	0.30	8.30 (1.15)	7.75 (1.40)	**0.24**
Mean blood glucose (d6)	9.73 (2.54)	**8.84 (2.23)**	0.12	10.02 (2.83)	8.69 (2.59)	**0.18**
Insulin use	27 (77)	**22 (61)**	0.20	11 (73)	11 (69)	**1.0**

## Data Availability

We did not obtain consent or ethical approval to share data in this way.
